# BEYOND FALLS: HOW DO FALL PREVENTION PROGRAMS BENEFIT THE COMMUNITY?

**DOI:** 10.1093/geroni/igae098.2858

**Published:** 2024-12-31

**Authors:** Jess VanderWerf

**Affiliations:** University of South Florida, Tampa, Florida, United States

## Abstract

This mixed methods research investigates the Co-Responder Program in Marion County and the CARES program in Pinellas County, Florida. It assesses their effectiveness in reducing fall-related injuries and mortality among older adults by analyzing community surveys administered to frequent 911 callers who subsequently participated in these programs and data from the counties and the state. The study explores the broader benefits of these programs beyond fall prevention, including their impact on reducing 911 calls and alleviating strain on community resources. In Pinellas County, about 65.22% of participants in the CARES program didn’t make any 911 calls post-enrollment, showcasing its effectiveness. Among those who still made calls, there was a 25.43% reduction in total calls. In Marion County, a sub-sample of 12 individuals used 1,330 hours of the sheriff’s office time for non-emergencies in 2021. Through the Co-Responders Program, there was an impressive 54% decrease in calls from these individuals. Further research is needed to understand the long-term impacts of these programs for the aging population while also standardizing what is and isn’t needed in one of these programs for effectiveness. The implications of this research show a growing need to expand training for first responders as it relates to aging issues as it relates to the potential for education in this research. These programs hold the potential to be implemented nationwide, thus freeing up community resources. However, to realize this future policy direction, a deeper understanding of these programs is imperative.

